# Lead Advancement Using Only a Stylet-Driven Lead and Stylet Without a Delivery Catheter: Aiming for Left Bundle Branch Area Pacing Without a Guiding Catheter

**DOI:** 10.7759/cureus.91627

**Published:** 2025-09-04

**Authors:** Daisuke Yamazaki, Naoto Yasuda, Kazuki Sato

**Affiliations:** 1 Cardiology, Akita Cerebrospinal and Cardiovascular Center, Akita, JPN; 2 Clinical Engineering, Akita Cerebrospinal and Cardiovascular Center, Akita, JPN

**Keywords:** body turn, left bundle branch area pacing, pacemaker implantation, stylet, stylet-driven pacing leads

## Abstract

Background: Left bundle branch area pacing (LBBAP) using a delivery catheter has become popular for achieving physiologic pacing, since lead advancement requires backup support, which is usually provided by a delivery catheter. However, traditional stylet shaping offers the advantage of flexible pacing site selection. Therefore, in this study, we investigated whether LBBAP can be performed using traditional stylet shaping.

Methods: We retrospectively reviewed 12 cases at our institution that underwent pacemaker implantation without using a delivery catheter, aiming to achieve LBBAP. Right ventricular (RV) angiography was performed, and the pacing site was selected using a conventional three-dimensional (3D) shaping stylet in the central region of the right ventricle, which was divided into nine segments, targeting a stimulated left ventricular activation time (LVAT) ≤ 80 ms. Next, we switched to a two-dimensional (2D) shaping stylet to achieve passive backup and performed lead advancement by rotating the lead body without pushing the stylet. We compared the LVAT, QRS width, and lead parameters over time.

Results: LVAT was significantly shortened from helix deployment (77.2 ± 10.7 ms) to body turn (71.5 ± 13.2 ms) (p = 0.00016) and further shortened by one week (62.5 ± 13.6 ms) (p = 0.0022).

Conclusions: By modifying the stylet-shaping curve and technique, lead advancement into the myocardium was feasible without using a delivery catheter. Although this study did not meet the strict criteria for LBBAP, our findings suggest that catheter-free LBBAP may be achievable with refined stylet shaping and standardized techniques.

## Introduction

In the early days of pacemaker implantation, tined leads were the only available option. The ventricular leads were placed at the apex, as it was the most accessible anatomical site. However, as the frequency of apical pacing increased, left ventricular contraction dyssynchrony was reported, resulting in decreased systolic function and atrial fibrillation [1,2]. With the availability of stylet-driven leads, it has become possible to shape the curve of the stylet and select pacing sites more flexibly, thereby achieving more physiologic conduction and ventricular synchrony. Consequently, right ventricular septal (RVS) pacing has become widely used. Recently, His bundle pacing (HBP) has been attempted to achieve physiological conduction and narrow QRS width; however, issues such as increased pacing thresholds and technical difficulties have been reported [3]. Subsequently, left bundle branch area pacing (LBBAP) was developed, in which the tip of a lumen-less lead is advanced into the interventricular septum to capture the left bundle branch [4]. Although it is not as effective as HBP in terms of physiological pacing, it is easier to perform and has a relatively narrow QRS width. This has led to its increasing adoption in recent years.

Until now, only the helix of stylet-driven leads has been directly screwed into the myocardium. However, LBBAP requires the tip of the lead body to be advanced into the myocardium, so pressure must be applied to the lead. Therefore, LBBAP requires a delivery catheter. The delivery catheters used for the lumen-less lead include the C315™ delivery catheter (Medtronic, Columbia, U.S.), the Site Selective Pacing Catheter (SSPC™) (Boston Scientific, Massachusetts, U.S.), the Selectra 3D™ catheter (Biotronik, Berlin, Germany), and the CPS Locator 3D™ (Abbott, Illinois, U.S.). High pressure can be applied to the RVS using a delivery catheter [5], and the lead can be turned clockwise to advance the lead tip into the myocardium to perform LBBAP. Because delivery catheters are ready-made products with a fixed shape, in some cases, they may not be able to be guided to a pacing site suitable for LBBAP. The use of a conventional stylet is advantageous in terms of the freedom to select the pacing site.

In this study, we investigated whether lead advancement and LBBAP could be performed as part of routine pacemaker implantation procedures using only a stylet-driven lead and stylet without the use of a delivery catheter.

## Materials and methods

We retrospectively reviewed patients who underwent pacemaker implantation without the use of a delivery catheter at Akita Cerebrospinal and Cardiovascular Center, in Akita, Japan, between June 2024 and March 2025. The inclusion criteria were cases with implanted ventricular leads, while the exclusion criteria were patients with renal dysfunction that precluded the use of contrast. No cases utilized a delivery catheter during this period, and no cases were excluded. During pacemaker implantation, the ventricular lead was placed with the aim of achieving LBBAP. The procedure is described below.

**Figure 1:**
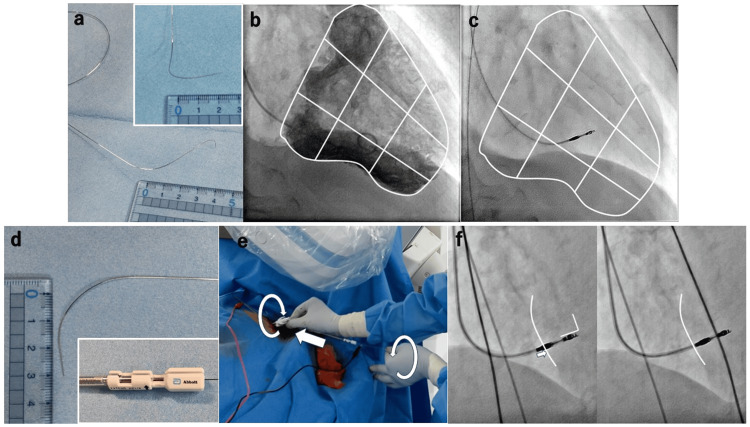
Right ventricular (RV) lead placement method. (a) Three-dimensional (3D) shaping of the stylet. The first curve is bent backward by approximately 2 cm to direct the lead tip toward the ventricular septum (inset). The second curve adjusts the pacing site height. (b) Right ventricular (RV) angiogram at right anterior oblique (RAO) 30°. The right ventricle is divided into nine sections. (c) RV lead placement. The right ventricle is divided into nine sections, with the central area as the index. The goal is to achieve a stimulated left ventricular activation time (LVAT) of 80 ms or less. (d) The initial stylet is replaced with a firm stylet shaped backward by approximately 3 cm to facilitate body turn and lead advance. The inset shows the Helix Locking Tool (Abbott, Illinois, U.S.). (e) Lead advance. The stylet is not allowed to move with applied counterclockwise torque; the lead is advanced along the stylet with applied clockwise torque. (f) Left anterior oblique (LAO) view before the body is turned on the left and after the lead is advanced during the body turn on the right. The white line in the right ventricular septum indicates the extent of lead advancement as shown by the arrow.

After local anesthesia, a pocket was created in the chest, and a sheath was inserted through the axillary vein. Right ventricular (RV) angiography was performed using a 4-French pigtail catheter (Outlook™, TERMO, Tokyo, Japan) in two directions: right anterior oblique (RAO) 30° and left anterior oblique (LAO) 60°. The catheter bed was kept stationary. The stylet was shaped in two curves: the first curve was bent backward (~2 cm) so that the tip faced the right ventricular septum (RVS), and the second curve was bent into a J-shape. The pacing site position was adjusted by modifying the J-curve (Figure 1a). As an indicator for LBBAP, the central area of the right ventricle, divided into nine sections in the RAO 30° view, was used as a reference (Figure 1b). A stylet-driven lead was guided to achieve a stimulated left ventricular activation time (LVAT) of ≤ 80 ms in lead V6 (Figure 1c). After removing the stylet, a firm stylet bent backward (~3 cm at the tip) was reintroduced, and the Helix Locking Tool (Abbott, Illinois, U.S.) was attached to the lead tail (Figure 1d). Counterclockwise torque was applied to the stylet so that the tip of the lead was perpendicular to the RVS, and the lead was then rotated clockwise under LAO fluoroscopy to advance it into the myocardium (Figure 1e). The endpoint of ventricular lead placement was an LVAT < 80 ms or a shortened LVAT achieved via body turn. The LAO view was used to compare the lead tip’s position before and after the lead advance to assess how far it had advanced. We recorded the current of injury during pacing in all cases and monitored resistance to ensure it remained ≥ 450 Ω to prevent microperforation. In cases of dual-chamber pacemaker implantation, the atrial lead was placed, the generator was connected, and the incision was closed to complete the procedure. The method for placing the ventricular lead is also shown in Video 1. QRS width, LVAT, R amplitude of the ventricular lead, threshold, and resistance were measured when the ventricular lead helix was deployed, after body turn, and at one week and one month later, and these were used as endpoints. This study was conducted in accordance with the Declaration of Helsinki. The ethics committee at our institution approved this study.

**Video 1:** Procedure for right ventricular (RV) lead placement. To avoid prolonging the manual procedure time, the lead was placed using the center of the RV angiography nine-segment division as a reference point, without targeting the qR or rsR waveform in the V1 lead. Therefore, the criteria for left bundle branch area pacing were not met.

Statistical analyses

All data were analyzed using EZR software (Saitama Medical Center, Jichi Medical University, Japan) based on R and R Commander (R Foundation for Statistical Computing, Vienna, Austria) [6]. Continuous variables were tested for normality using the Kolmogorov-Smirnov test. Normally distributed variables were presented as mean ± standard deviation, while non-normally distributed variables were presented as median (interquartile range). Comparisons between groups were performed using the paired t-test for normally distributed data and the Wilcoxon signed-rank test for non-normally distributed data. A p-value < 0.05 was considered statistically significant in all analyses.

## Results

A total of 12 pacemaker implantations were performed between June 2024 and March 2025. Table 1 presents the details of the cases. The mean age of the patients was 77.0 ± 8.8 years, and three cases included additional ventricular leads during pacemaker generator replacement. The ventricular leads used included Tendril™ STS (Abbott, Illinois, U.S.), CapSureFix Novus MRI™ (Medtronic, Columbia, U.S.), INGEVITY™ (Boston Scientific, Massachusetts, U.S.), and VEGA™ (Micro Port, Tennessee, U.S.), among others. The average procedure time was 104.0 ± 17.2 minutes, and care was taken to ensure that the procedure time did not exceed two hours. The LVAT was 77.2 ± 10.7 ms during helix deployment and 71.5 ± 13.2 ms after body turn. The mean QRS time after body turn was 136.7 ± 15.8 ms. The mean R wave amplitude and capture threshold after pacemaker implantation were 8.3 ± 4.2 mV and 0.7 ± 0.3 V, respectively, and were stable. One case showed a qR pattern in lead V1.

**Table 1:** Patient backgrounds and procedure outcomes. AV: atrioventricular; SSS: sick sinus syndrome; RV: right ventricular; LVAT: left ventricular activation time

Case no.	Age	Sex	Arrhythmias	RV lead	Procedure time (min)	Fluoroscopic time (min)	After screw LVAT (ms)	After screw QRS (ms)	Body turn LVAT (ms)	Body turn QRS (ms)	R wave amplitude (mV)	Capture threshold (V)	Impedance (Ω)
1	87	Male	Complete AV block	Tendril™ STS 52 cm	107	26	64	126	54	112	5	1	552
2	85	Female	SSS	Tendril™ STS 52 cm	130	32	-	-	87	138	5	0.5	450
3	74	Female	SSS	Tendril™ STS 52 cm	93	17	67	-	58	148	>12	0.75	510
4	73	Male	SSS	CapSureFix Novus MRI™ 52 cm	110	13	81	-	72	140	3.1	0.5	570
5	84	Male	Trifascicular block	Tendril™ STS 52 cm	90	17	81	135	74	135	3.9	1.3	658
6	77	Male	Complete AV block	Tendril™ STS 52 cm	128	30	83	-	71	152	-	1	530
7	66	Female	SSS	Tendril™ STS 52 cm	95	17	83	131	78	131	>12	0.75	580
8	85	Female	SSS	Tendril™ STS 52 cm	115	13	65	82	53	111	10.9	0.4	546
9	61	Male	Complete AV block	INGEVITY™ 52 cm	100	22	94	162	87	155	-	0.7	1000
10	87	Female	SSS	INGEVITY™ 52 cm	80	10	83	127	81	120	10	0.4	750
11	78	Male	SSS	VEGA™ 52 cm	80	12	62	132	56	140	15.2	0.5	638
12	68	Male	SSS	Tendril™ STS 52 cm	120	24	86	158	87	158	5.5	0.8	643

**Figure 2:**
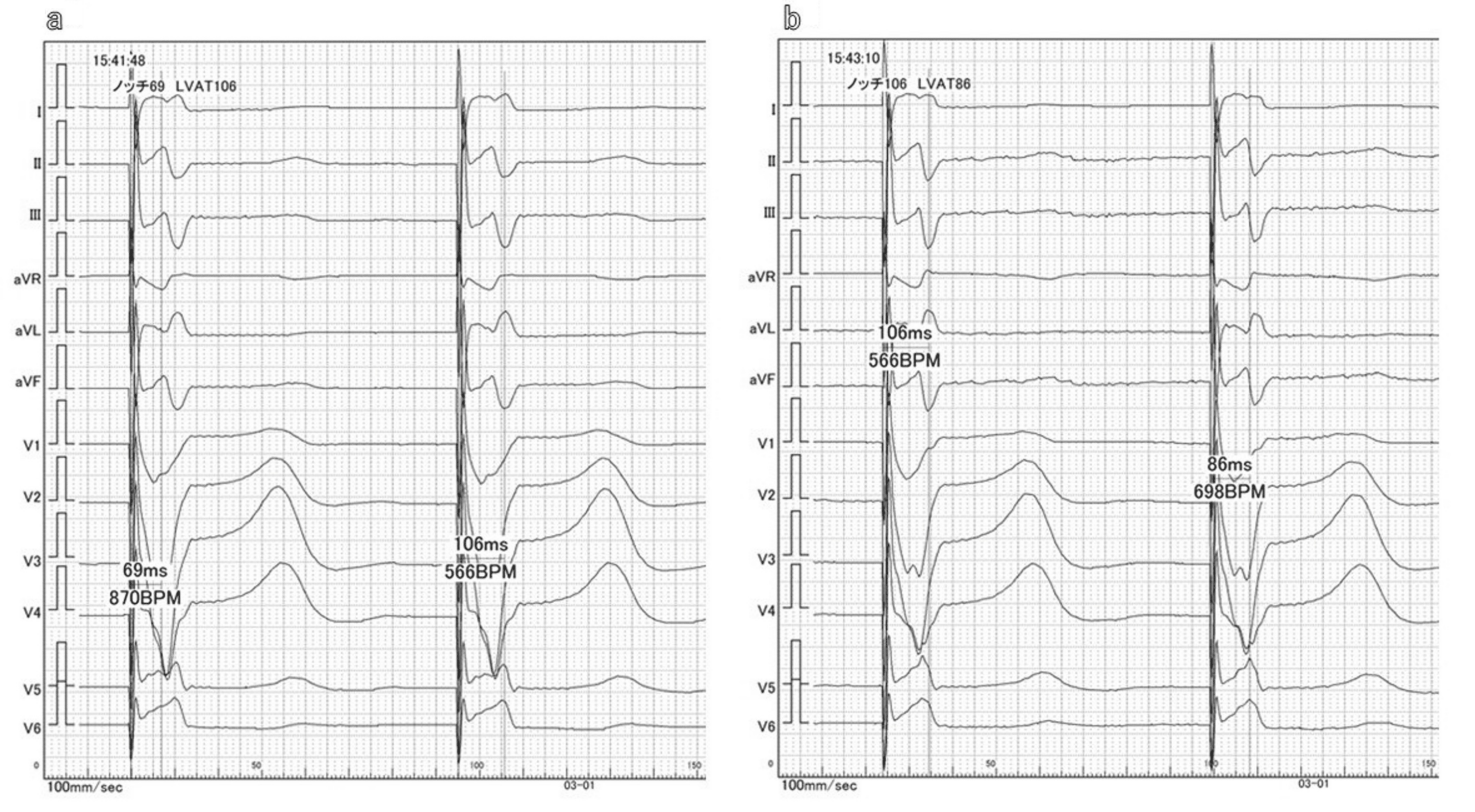
Example of an electrocardiogram (ECG) during a procedure (100 mm/s). (a) ECG at helix deployment, in which the time from pacing to V1 notch was 69 ms, and the V6 stimulated left ventricular activation time (LVAT) was 106 ms. (b) ECG after lead advance, in which the time from pacing to V1 notch was moved backward to 106 ms, and the V6 LVAT was shortened to 86 ms, suggesting that lead advance was achieved.

Figure 2 shows a typical example of the electrocardiogram (ECG) during implantation. Figure 2a shows the ECG during lead helix deployment, and Figure 2b shows the ECG after lead advance. It can be seen that the notch moved backward in V1 and the LVAT shortened in V6. If the lead advance had been slightly advanced, the qR waveform could have been observed in the V1 lead.

**Figure 3:**
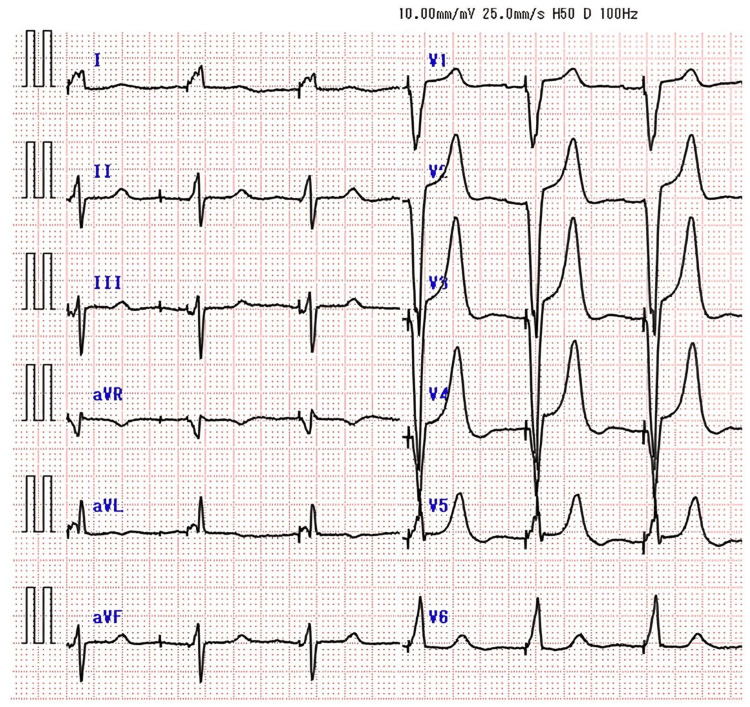
A post-implantation electrocardiogram (ECG). In this study, we did not aim for qR or rsR in the V1 lead because of concerns about prolongation of procedure time. Therefore, it is difficult to say that this is a typical ECG waveform of left bundle branch area pacing.

Figure 3 shows a typical post-implantation ECG. Since the endpoint of the procedure in this study was the shortening of LVAT, few cases achieved qR or rsR waveforms in V1 induction; however, a notch was observed backward.

**Figure 4:**
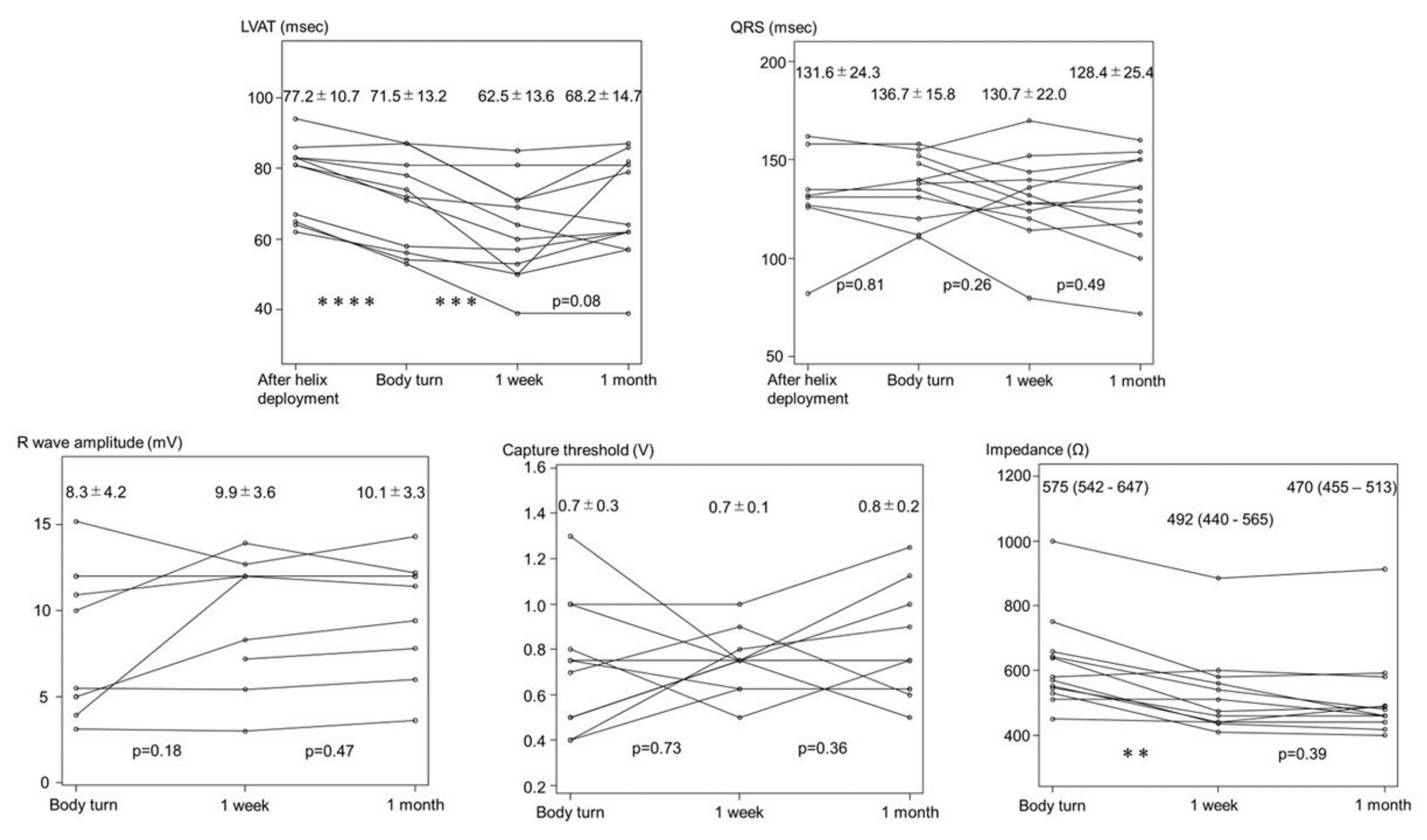
Changes in various parameters. The graphs show the changes in left ventricular activation time (LVAT) and QRS width from the time of helix deployment to one month post-pacemaker implantation and the changes in R wave amplitude, capture threshold, and impedance from the time of lead body turn to one month post-pacemaker implantation. Intergroup comparisons were performed for each parameter at each follow-up. ** p < 0.01, *** p < 0.005, **** p < 0.001.

Figure 4 shows the changes in LVAT and QRS width after helix deployment, body turn, one week, and one month, and the changes in R wave amplitude, capture threshold, and impedance after body turn, one week, and one month. Regarding LVAT, there was a significant reduction from after helix deployment (77.2 ± 10.7 ms) to after body turn (71.5 ± 13.2 ms) (p = 0.00016), and a significant reduction from after body turn to one week later (62.5 ± 13.6 ms) (p = 0.0022). There was no significant difference in the QRS width between one month and one week after the helix deployment. Regarding the pacemaker lead parameters, impedance decreased significantly (p = 0.0067) from after body turn (575 (542 - 647) Ω) to one week later (492 (440 - 565) Ω). No complications were observed in these 12 cases.

## Discussion

With the availability of stylet-driven leads, RVS pacing, which is closer to the physiological conduction system than apex pacing, has become possible. RVS pacing reduces the incidence of heart failure progression and mortality compared with apex pacing [7], and it has become widely used. Subsequently, HBP was attempted with the expectation of further physiological stimulus conduction and left ventricular contraction synchrony [8]. At that time, various delivery catheters were developed by different companies, and HBP was attempted; however, difficulties in the procedure and a tendency toward high pacing thresholds prevented it from becoming as widely used as RVS pacing. Given this background, LBBAP has been adopted with the expectation of achieving stable success rates by advancing the lead tip into the myocardium using a delivery catheter and aiming to capture the left bundle branch [4]. Although LBBAP does not achieve QRS narrowing comparable to HBP, it is easier to perform and has recently attracted attention.

The criteria for LBBAP have not yet been established, but the following are currently used as guidelines: (i) paced QRS morphology with a right bundle branch block (RBBB)-like pattern with an rsR or qR pattern; (ii) recording of a left bundle branch potential; (iii) abrupt shortening of R wave peak times in V6 by > 10 ms at high output pacing during lead advancement from the midseptal region to the left ventricular endocardium; (iv) short LVAT (usually ≤ 80 ms) at both high and low output; and (v) demonstration of selective capture. Many studies consider LBBAP achieved if criterion (i) and at least one other criterion are met [9-11]. LBBAP methods include (i) targeting 1-1.5 cm apical to the site where HBP is achieved [12]; (ii) dividing the contrast-enhanced RV into nine segments and targeting the center [13]; and (iii) dividing the RV silhouette into nine segments without contrast enhancement and targeting the center [14].

**Figure 5:**
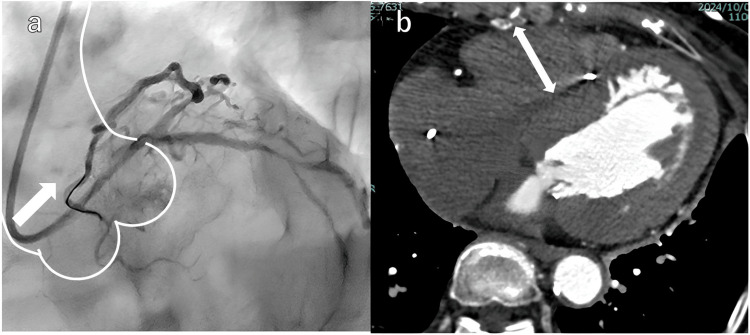
Illustrations showing the guiding catheter obtaining backup during percutaneous coronary intervention (PCI) and the explanation for why the stylet was bent approximately 3 cm backward. (a) Spider view. PCI using a guiding catheter (TAIGA™ EBU3.5; Medtronic, Columbia, U.S.). The guiding catheter is stable during treatment because it contacts the aortic wall and passive backup is applied (arrow). (b) Horizontal slice at the cardiac cavity level on contrast-enhanced computed tomography (CT). The width of the right ventricle is approximately 3 cm (arrow); thus, the stylet used for the lead advance was shaped backward by approximately 3 cm.

LBBAP is generally performed using a delivery catheter; however, satisfactory endpoints may not be achieved in some cases because delivery catheters have a predetermined shape. A delivery catheter is widely used because a backup force is required to advance the lead tip into the myocardium by turning the lead body; however, adjusting the stylet with a shaping curve allows for greater flexibility in selecting the pacing site. Therefore, we investigated whether it was possible to advance the lead into the myocardium by modifying the shaping curve of the stylet. In the early stages of verification, we tried to advance the lead with the stylet in the shaping of the stylet (Figure 1a) that is often done in conventional RVS pacing [15]. However, applying the weight perpendicularly to the RVS was difficult. Subsequently, we tried to add weight to the RVS by pushing only the lead - without pushing the stylet - while keeping the stylet fixed in the same position. However, there was strong resistance to advancing only the lead along the three-dimensional (3D) stylet shaping, and there was a time lag in transferring the torque from the body turn to the lead tip. The next method we developed - and the one used in this study - was inspired by the guiding catheter used in percutaneous coronary intervention (PCI). A border-type guiding catheter obtains passive backup on the opposite side of the aorta or aortic valve during PCI of the left coronary artery (Figure 5a). We hypothesized that it may be possible to obtain passive backup on the RV anterior wall in the same manner. This led to the two-dimensional (2D) stylet shaping used in this method.

Until the lead helix was screwed into the myocardium, the pacing site was adjusted using the conventional 3D stylet-shaping method, which facilitated the adjustment. When the lead body was turned, the stylet was replaced with a 2D curve stylet to provide passive backup. Generally, the distance from the ventricular septum of the pacing site to the RV anterior wall was approximately 3 cm (Figure 5b). We considered that bending the stylet tip backward by approximately 3 cm would be appropriate for obtaining passive backup of the RV anterior wall. After inserting the 2D curve stylet, counterclockwise torque was applied to make the lead tip perpendicular to the RVS. While maintaining this situation, the fluoroscopy detector was set to LAO, and only the lead was turned to advance it into the myocardium. The lead tip was advanced along the curve of the stylet, and we thought that this would allow us to add pressure to the RVS in order to obtain passive backup of the RV anterior wall. Additionally, because the stylet had a 2D curve, there was less resistance than the 3D stylet shaping used when screwing in the lead helix, making it easier to advance the lead along the stylet. A significant decrease in ventricular lead resistance and LVAT was observed from one week after pacemaker implantation, which may have been due to residual torque from the lead advance. However, there were only a few cases in which the lead resistance fell below 450 Ω, suggesting that safety was guaranteed.

Similar to our findings, Ludwik et al. performed LBBAP without using a delivery catheter [16]. The sample size was large, and qR or rsR waveforms in the V1 leads were clear in most cases, indicating that LBBAP was performed appropriately. Lead advancement initially involved shaping the stylet into a 3D swan-neck configuration. If unsuccessful, the stylet curve was modified into alternative shapes, resembling several types of commercially available delivery catheters. In our study, we used two types of stylet curves from the start: the conventional 3D swan-neck shape for the helix screw-in phase, and then switched to a 2D stylet curve to achieve passive backup, which is a novel approach and the first report of this kind.

Unlike when using a delivery catheter, catheter tip angiography was not feasible. Therefore, the fluoroscopic image at the start of the body turn in the LAO view was saved and used as a reference to confirm that the lead tip was advancing (Figure 1e). In this study, LVAT was significantly shorter after the body turn than after screw-in, indicating that lead advancement into the myocardium can be achieved without a delivery catheter. However, without tip angiography, the exact depth of lead penetration could not be determined. Moreover, due to concerns about ventricular septal perforation, changes in the V1 lead of the ECG were not attempted. Therefore, the excellent endpoints typically achieved with LBBAP using a delivery catheter were not achieved. Our institution has not performed many pacemaker implantations, and we are still learning the LBBAP technique. Based on the LBBAP indicators and methods described above, we aimed to perform LBBAP. However, because we anticipated that strict adherence to the indicators would prolong the procedure time while we were still unfamiliar with the LBBAP, we adopted the following simple indicators for cases during this period: (i) target the central portion of the right ventricle divided into nine segments as visualized by angiography, and (ii) achieve a short LVAT (usually ≤ 80 ms) at low output. Therefore, LBBAP cannot be strictly considered in this study. However, LVAT ≤ 80 ms was generally achieved, and a significant reduction in LVAT was confirmed with the lead body turn, suggesting that lead advance was possible with the stylet alone. The limitation of lead advancement using only the stylet is that it is not possible to confirm the number of millimeters the lead tip has advanced, as is possible with a delivery catheter. In this study, contrast injection was performed only during the initial RV angiography, and the tube angle was set to RAO 30° and LAO 60°. Since the position of the heart varies slightly depending on the body type of each case, the observation of the lead tip from the side at LAO 60° may not always be possible. In this study, due to the aforementioned reasons, including the difficulty in determining the distance of lead tip advance, the lack of experience with LBBAP at our institution, and concerns about ventricular septal perforation, we were unable to actively advance the lead compared to lead advance using a delivery catheter, resulting in incomplete LBBAP. The main point emphasized in this study is that we have demonstrated the possibility of performing LBBAP using a conventional stylet, which is currently the mainstream method using a delivery catheter.

Limitations

This study had a small sample size and was a single-group retrospective study that used multiple types of ventricular leads. LVAT shortening alone cannot confirm left bundle branch capture, and the follow-up period was only one month, which are limitations of this study.

## Conclusions

In conclusion, modifying the stylet shape and refining the technique allows for lead advancement into the myocardium without a delivery catheter. Furthermore, standardizing the pacing site and refining lead advancement may enable LBBAP without using a delivery catheter.

## References

[1] Thambo JB, Bordachar P, Garrigue S (2004). Detrimental ventricular remodeling in patients with congenital complete heart block and chronic right ventricular apical pacing. Circulation.

[2] Sweeney MO, Hellkamp AS, Ellenbogen KA, Greenspon AJ, Freedman RA, Lee KL, Lamas GA (2003). Adverse effect of ventricular pacing on heart failure and atrial fibrillation among patients with normal baseline QRS duration in a clinical trial of pacemaker therapy for sinus node dysfunction. Circulation.

[3] Vijayaraman P, Naperkowski A, Subzposh FA (2018). Permanent His-bundle pacing: long-term lead performance and clinical outcomes. Heart Rhythm.

[4] Zhang S, Zhou X, Gold MR (2019). Left bundle branch pacing: JACC review topic of the week. J Am Coll Cardiol.

[5] Yamazaki D (2024). Measurement of tip load with a pacemaker lead stylet and guiding catheter using a silicone heart model. Cureus.

[6] Kanda Y (2013). Investigation of the freely available easy-to-use software 'EZR' for medical statistics. Bone Marrow Transplant.

[7] Kikuchi M, Tanno K, Miyoshi F (2012). Long-term effectiveness of right septal pacing vs. right apical pacing in patients with atrioventricular block. J Arrhythmia.

[8] Abdelrahman M, Subzposh FA, Beer D (2018). Clinical outcomes of His bundle pacing compared to right ventricular pacing. J Am Coll Cardiol.

[9] Liu X, Niu HX, Gu M (2021). Contrast-enhanced image-guided lead deployment for left bundle branch pacing. Heart Rhythm.

[10] Li Y, Chen K, Dai Y (2019). Left bundle branch pacing for symptomatic bradycardia: implant success rate, safety, and pacing characteristics. Heart Rhythm.

[11] Chen K, Li Y (2019). How to implant left bundle branch pacing lead in routine clinical practice. J Cardiovasc Electrophysiol.

[12] Huang W, Su L, Wu S, Xu L, Xiao F, Zhou X, Ellenbogen KA (2017). A novel pacing strategy with low and stable output: pacing the left bundle branch immediately beyond the conduction block. Can J Cardiol.

[13] Jiang H, Hou X, Qian Z (2020). A novel 9-partition method using fluoroscopic images for guiding left bundle branch pacing. Heart Rhythm.

[14] Zhang J, Wang Z, Zu L (2021). Simplifying physiological left bundle branch area pacing using a new nine-partition method. Can J Cardiol.

[15] Burri H, Domenichini G, Sunthorn H, Ganière V, Stettler C (2012). Comparison of tools and techniques for implanting pacemaker leads on the ventricular mid-septum. Europace.

[16] Ludwik B, Labus M, Roleder T, Moskal P, Kiełbasa G, Śpikowski J, Jastrzębski M (2024). Novel approach to left bundle branch area pacing lead implantation using a 3-dimensional stylet. Heart Rhythm.

